# Electroacupuncture attenuated cerebral ischemic injury and neuroinflammation through α7nAChR-mediated inhibition of NLRP3 inflammasome in stroke rats

**DOI:** 10.1186/s10020-019-0091-4

**Published:** 2019-05-22

**Authors:** Tao Jiang, Meiyan Wu, Zhanqin Zhang, Chaoying Yan, Zhi Ma, Shan He, Wei Yuan, Kairui Pu, Qiang Wang

**Affiliations:** Department of Anesthesiology, Center for Brian Science, The First Affiliated Hospital of Xi’an Jiaotong University, Xi’an, 710061 Shaanxi Province China

**Keywords:** Electroacupuncture, α7nAChR, NLRP3 inflammasome, Cerebral ischemia-reperfusion injury

## Abstract

**Background:**

Our previous research confirmed that electroacupuncture (EA) stimulus elicits neuroprotective effects against cerebral ischemic injury through α7 nicotinic acetylcholine receptor (α7nAChR)-mediated inhibition of high-mobility group box 1 release mechanism. This study investigated whether the signal transducer of α7nAChR and inhibition of NLRP3 inflammasome are involved in the neuroprotective effects of EA stimulus.

**Methods:**

In adult male Sprague-Dawley rats, the focal cerebral ischemic injury was induced by middle cerebral artery occlusion (MCAO) models for 1.5 h. The expression of NLRP3 inflammasome in the penumbral tissue following reperfusion was assessed by western blotting and immunoflourescent staining. The infarct size, neurological deficit score, TUNEL staining and the expression of proinflammatory factors or anti-inflammatory cytokines were evaluated at 72 h after reperfusion in the presence or absence of either α7nAChR antagonist (α-BGT) or agonist (PHA-543,613).

**Results:**

The contents of inflammasome proteins were gradually increased after cerebral ischemia/reperfusion (I/R). EA stimulus attenuated NLRP3 inflammasome mediated inflammatory reaction and regulated the balance between proinflammatory factors and anti-inflammatory cytokines. The agonist of α7nAChR induced similar neuroprotective effects as EA stimulus. In contrast, α7nAChR antagonist reversed not only the neuroprotective effects, but also the inhibitory effects of NLRP3 inflammasome and the regulatory effects on the balance between proinflammatory factors and anti-inflammatory cytokines.

**Conclusions:**

These results provided compelling evidence that α7nAChR played a pivotal role in regulating the activation and expression of NLRP3 inflammasome in neurons after cerebral I/R. These findings highlighted a novel anti-inflammatory mechanism of EA stimulus by α7nAChR modulating the inhibition of NLRP3 inflammasome, suggesting that α7nAChR-dependent cholinergic anti-inflammatory system and NLRP3 inflammasome in neurons might act as potential therapeutic targets in EA induced neuroprotection against cerebral ischemic injury.

## Background

Stroke is the second leading cause of death and third leading cause of disability-adjusted life-years lost globally (Hankey 2017). Although r-tPA is considered to be an effective treatment strategy, only 3–5% of patients may benefit from it due to a relatively narrow therapeutic time window (Roger et al. 2012). Our team has proposed a new approach of electroacupuncture (EA) neuroprotection for the prevention of cerebral ischemia injury. Our previous studies have reported that EA could relieve neurological disorders, reduce infarct volumes after focal cerebral ischemia (Wang et al. 2009) and regulate the Reperfusion Injury Salvage Kinase (RISK) signaling pathway (ERKε, PKC, GSK-3β, STAT3) via cannabinoid receptor 1 (CB1R)(Wang et al. 2011) and inhibit neuronal apoptosis(Guo et al. 2015; Sun et al. 2016; Wei et al. 2014; Zhou et al. 2013). In addition, we demonstrated that EA could attenuate cerebral ischemic injury via regulation of α7nAChR-mediated inhibition of HMGB1 release in rats (Wang et al. 2012). Immunity and inflammation plays an integral part in the pathophysiology of stroke and is involved in all stages of ischemic cascade. EA has been endorsed by the World Health Organization and accumulating evidences from several clinical trials and other updated reviews supported its effectiveness in perioperative and stroke rehabilitation (Vickers et al. 2018). But the detailed underlying mechanism to regulate inflammation after ischemic stroke remains unclear (Goldman et al. 2010).

As an integrated platform for triggering inflammation, inflammasome contributes to the pathogenesis of initiating innate immune response after cerebral ischemia/reperfusion (I/R)(Ismael et al. 2018; Kerr et al. 2018; Ren et al. 2018). NLRP3 inflammasome is composed of NLRP3 (nucleotide-binding oligomerization domain (NOD)-like receptor (NLR) pyrin domain-containing 3), ASC (apoptosis-associated speck-like protein containing a caspase recruitment domain) and procaspase-1, subsequently amplifying the production and secretion of proinflammatory cytokines, apoptotic and pyroptotic cell deaths(Zhong et al. 2018). The activation of NLRP3 inflammasome is based on two functionally distinct steps i.e., ‘priming’ and ‘activation’ (Zhong et al. 2018). When all kinds of danger signals interact with TLRs on the cell membrane, NF-κB pathway is activated, promoting the synthesis of inflammasome proteins proIL-1β and proIL-18 (Fann et al. 2018; Ren et al. 2018). Moreover, the intracellular danger-associated molecular patterns (DAMPs) could oligomerize NLRP3 sensors and convert precursor caspase-1 into cleaved caspase-1 (Cl.Caspase-1). The cleaved caspase-1 can then promote the cleavage of precursors IL-1β, IL-18 and increase the release of biologically active mature IL-1β and IL-18. Furthermore, cleaved caspase-1 may trigger a form of proinflammatory programmed cell death, known as pyroptosis (Fann et al. 2018; Ren et al. 2018). It is characterized by rapid plasma membrane rupture, cellular swelling, release of pro-pyroptosis factors, DNA cleavage and nuclear condensation, based on cleaved caspase-1 through an unknown mechanism (Bergsbaken et al. 2009). The pore-forming protein gasdermin D (GSDMD) cleaved by caspase-1 was identified as the principal executioner of pyroptosis. Caspase-1 and GSDMD promotes the activation of IL-1β and IL-18 either directly or through a signaling cascade, leading to pyroptosis (McKenzie et al. 2018).

However, an inflammatory reflex of vagus nerve can suppress the release of cytokines in the nervous system (Bertrand et al. 2015; Han et al. 2017). The efferent neural signaling pathway is termed as the cholinergic anti-inflammatory pathway, inhibiting the synthesis of cytokines and preventing the damage effects of cytokines release during I/R injury, sepsis, arthritis and other inflammatory syndromes (Bernik et al. 2002; Borovikova et al. 2000; de Jonge et al. 2005; Guarini et al. 2003; Yu et al. 2013). As a target of cholinergic anti-inflammatory pathway, α7 nicotinic acetylcholine receptor (α7nAChR) plays a critical role in the transmission of cholinergic signal for the suppression of systemic inflammation (Borovikova et al. 2000; Wang et al. 2004; Wang et al. 2003). Our previous study has reported an association between α7nAChR and cerebral ischemia in middle cerebral artery occlusion (MCAO) rat model, where EA stimulus alleviates cerebral I/R injury through α7nAChR-mediated inhibition of HMGB1 release (Wang et al. 2012).

Therefore, the current study aimed to examine the hypothesis of whether EA attenuates cerebral I/R injury through α7nAChR-mediated inhibition of NLRP3 inflammasome caused inflammatory response to provide a novel therapeutic target for ischemic stroke and a remarkable theoretical basis for the neuroprotection of EA.

## Materials and methods

### Animals and drugs

The experimental protocol was approved by the Ethics Committee for Animal Experimentation. Sprague–Dawley (SD) rats, weighting 250-280 g were obtained from the Experimental Animal Centre of Fourth Military Medical University. The SD rats were maintained in a 12 h light–dark cycle at a temperature of 20–25 °C, with an air humidity of 60%, and had free access to food and water for at least 1 week. Rats received humane care in accordance with the Guidelines for Animal Experimentation of the Fourth Military Medical University, Xi’an, China. Great efforts have been made to minimize the number of animals.

**Fig. 1:**
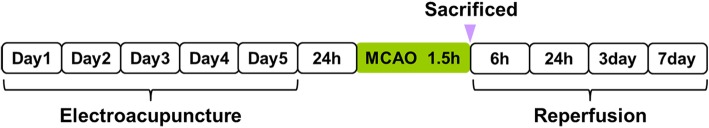
Experimental design. Electroacupuncture stimulus was given at a frequency of 2/15 Hz and an intensity of 1 mA for 30 min for 5 consecutive days. After 24 h of the last stimulus, the rats were subjected to MCAO for 1.5 h and were sacrificed for research at 6 h, 24 h, 3d, 7d after reperfusion

The specific α7nAChR antagonist α-bungarotoxin (α-BGT) was purchased from the American Alexis Biochemicals Corporation and was dissolved in 150 mM NaCl before using. As α-BGT cannot penetrate the blood-brain barrier, it was intracerebroventricularly injected 30 min prior to EA at a dose of 0.5 μg/kg for 5 consecutive days according to the previous study (Kempsill and Pratt 2000). The potent and selective agonist for α7nAChR, N-[(3R)-1-Azabicyclo[2.2.2]oct-3-yl]furo[2,3-c] pyridine-5-carboxamide hydrochloride (PHA-543,613 hydrochloride) was obtained from American Tocris Bioscience and it has a good blood-brain barrier permeability. It was dissolved in saline before intraperitoneal injection (1.0 mg/kg) for 5 days (Wishka et al. 2006). The experimental design was shown in Fig. 1.

### Intracerebroventricular injection

After fasting for 12 h and deprivation of water for 4 h, the SD rats underwent lateral ventricle catheter operation. Anesthetized rats were fixed in a stereotaxic apparatus (Narishige, Tokyo, Japan). The rats were exposed at the bregma point after separating the subcutaneous soft tissue and then wiped with hydrogen peroxide solution. The target point was located at 1.4 mm lateral and 0.8 mm posterior to the bregma point in the right hemisphere, and a 1 mm diameter holes (O point) were drilled by using a skull drill. In addition, another two points (A, B points) were drilled, respectively in the front-point and back-point of the skull. The three points constituted as an equilateral triangle. Then the lateral ventricle tube was merged with O point at a depth of about 2.6 mm to 2.8 mm and two small screws were fixed at points A and B. Dental glue was used to fix the three points, preventing the rats from pulling the lateral ventricle tube after awakening.

### Electroacupuncture stimulus

EA was performed according to the previously established protocol (Wang et al. 2012). The acupoint Baihui (GV20), which was located at the intersection of the sagittal midline and the earline of the rat, was stimulated by using a Hwato EA instrument (Model No. SDZV, Suzhou Medical Appliances Co, Ltd., Suzhou, China). The stimulated parameters were given at a frequency of 2/15 Hz and an intensity of 1 mA for 30 min for 5 consecutive days. After the last end of the last EA stimulus, the animals were subjected to MCAO models for 24 h. To maintain the core temperature of 37.0 ± 0.5 °C, a surface heating plate (Spacelabs Medical Inc., Redmond, WA) was used. Control group rats received only anesthesia without giving acupuncture stimulation.

### Middle cerebral artery occlusion model

The cerebral I/R injury was induced in male SD rats according to the previously described protocol (Wang et al. 2009). The rats were anesthetized with 3% isoflurane. After midline incision of the neck and dissociation of the subcutaneous soft tissue on to the right side, a heat blunted 3–0 nylon suture was used to obstruct the middle cerebral artery (MCA) though the right common carotid artery and the internal carotid artery. The suture was permitted to remain in position for 1.5 h, and then was removed for reperfusion. Transcranial laser Doppler flowmetry (PeriFlux 5000, Perimed AB, Sweden) was performed to measure the regional cerebral blood flow. The rats in the Sham group underwent similar protocol without inserting the suture.

### Neurobehavioral evaluation and measurement of infarct size

Neurobehavioral evaluation was performed on day 3 after reperfusion by a blinded observer in accordance with an 18-point neurobehavioral scoring system, which contains side stroking, limb symmetry, spontaneous activity, vibris touch, forelimb walking and climbing (Garcia et al. 1995). The brains were then removed quickly from the deeply anesthetized rats and the infarct volume was measured as previously described (Wang et al. 2009). Six slices were stained with 2% 2,3,5-triphenyltetrazolium chloride (TTC, Sigma-Aldrich, St. Louis, MO) at 37 °C for 20 min followed by immersion in 4% paraformaldehyde for 24 h. After that, the slices were photographed with a camera (Canon G11, Canon Inc., Japan) and the infarct sizes were measured by image analysis software (Adobe Photoshop CS3 Extended, USA). The evaluation of a relative infarct size was made according to the following equation: relative infarct size = (contralateral area − ipsilateral non-infarct area) / contralateral area.

### Western immunoblot

According to the established protocol, the brain tissue corresponding to the ischemic penumbra was harvested (Wei et al. 2014). The coronal brain slice was homogenized in ice-cold RIPA lysis buffer (Beyotime, Nantong, China), and then was added to 1 × Roche complete protease inhibitor cocktail. The protein concentration was determined by Bradford method. Western immunoblotting was conducted in accordance with the previously described protocol (Wei et al. 2014). Primary antibodies used in this study were as follows: anti-NLRP3 rabbit polyclonal antibody, anti-α7nAChR rabbit polyclonal antibody (both used at a dilution of 1:500, Santa Cruz Biotechnology, USA), and anti-Caspase-1 rabbit polyclonal antibody, anti-IL-1β polyclonal antibody (both used at a dilution of 1:1000, Santa Cruz Biotechnology, USA), anti-GSDMD rabbit monoclonal antibody (1:1000 dilution, abcam, USA), GAPDH monoclonal antibody (1:1000 dilution, CWBIO, China), and β-tubulin monoclonal antibody (1:1000 dilution, CWBIO, China). The membranes were then incubated with appropriate secondary antibodies for 2 h including horseradish peroxidase-conjugated goat anti-rabbit (used at a dilution of 1:20,000, GSGB-BIO, China).

### TUNEL staining

Quantitative TUNEL staining was conducted as reported previously for detecting the apoptosis of neuronal cells in the ischemic penumbra (Wang et al. 2011). The tissues were fixed in 4% paraformaldehyde on day 3 after reperfusion. Under Olympus fluorescence microscope, 32 pixels of 0.10 mm^2^ were located by using a × 100 magnification objective lens. In the pixels, the total number of positively stained cells was counted and expressed as cells per square millimeter.

### Immunoflourescent staining

After preparing the frozen sections, the ischemic penumbral brain tissues were cut into 12-μm thick coronal sections on a cryostat. NLRP3 labeling was performed by incubating the sections with primary anti-NLRP3 rabbit monoclonal antibody (1:50 dilution; Santa Cruz Biotechnology, USA) and primary anti-mouse Neuronal Nuclei polyclonal antibody (NeuN, 1:500; Millipore) for overnight at 4 °C. Caspase-1 labeling was performed by incubating the sections with primary anti-Caspase 1 rabbit monoclonal antibody (1:50 dilution; Santa Cruz Biotechnology, USA) and primary anti-mouse Neuronal Nuclei polyclonal antibody (NeuN, 1:500; Millipore) for overnight at 4 °C. α7nAChR labeling was performed by incubating the sections with primary anti-α7nAChR rabbit monoclonal antibody (1:50 dilution; Santa Cruz Biotechnology, USA) and primary anti-mouse neuronal nuclei polyclonal antibody (NeuN, 1:500; Millipore) for overnight at 4 °C. The sections were then washed thrice with PBS (phosphate-buffered saline) and incubated with green-fluorescent Alexa Fluor 488 and red-fluorescent Alexa Fluor 594 (both used at a dilution of 1:500; Life Technologies, USA) for 4 h at room temperature. The sections were observed and the images were captured subsequently by using a confocal fluorescence microscopy (BX51, Olympus, Tokyo, Japan).

#### Elisa

Brain tissues corresponding to the ischemic penumbra were harvested according to the established protocol (Sun et al. 2016). The measurement of pro-inflammatory factors (IL-18, TNF-α) and anti-inflammatory factors (TGF-β1, IL-10) was performed by ELISA kits according to the manufacturer’s instructions.

### Statistical analysis

The SPSS 18.0 software was used for statistical analysis. Except for the neurological scores, all values were expressed as mean ± SD and analyzed by one-way ANOVA. The median ± interquartile range was used to describe neurologic deficit scores, followed by analysis by using Kruskal–Wallis test, Mann–Whitney *U* test and Bonferroni post hoc correction. *P* < 0.05 was considered to be statistically significant.

## Results

### Cerebral ischemia-reperfusion injury increased the expression of NLRP3 inflammasome proteins in rats

**Fig. 2:**
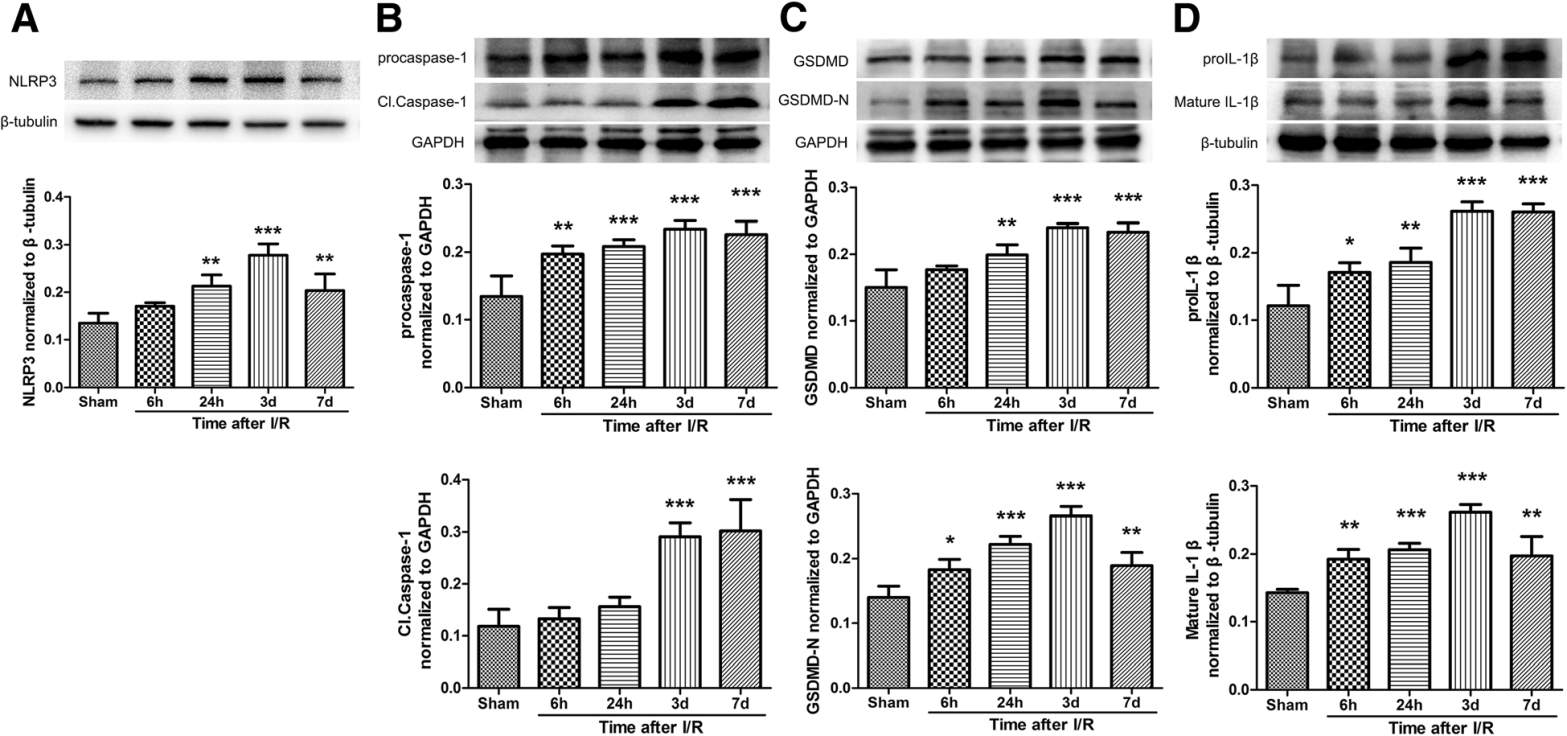
Effect of cerebral ischemia-reperfusion injury on the expression of NLRP3 inflammasome. Representative western immunoblotting analysis of NLRP3 (**a**), Caspase-1 (**b**), GSDMD (**c**) and IL-1β (**d**) (*n* = 4). Cerebral ischemia/reperfusion injury significantly increased NLRP3 inflammasome levels such as NLRP3, procaspase-1, Cl.Caspase-1, GSDMD, GSDMD-N, proIL-1β and mature IL-1β. The contents showed the highest at 3 days after reperfusion. *P < 0.05, **P < 0.01, ***P < 0.001 vs. Sham group

To observe the expression of NLRP3 inflammasome at different time points after cerebral I/R, rats were subjected to MCAO for 90 min. At 6 h, 24 h, 3 days and 7 days after reperfusion, the rats were sacrificed and the penumbral tissues were evaluated to analyze the levels of NLRP3 inflammasome (NLRP3, Caspase-1 and IL-1β). Compared with Sham group, the contents of NLRP3 at 24 h (*Ρ* < 0.01), 3 days (*Ρ* < 0.001), and 7 days (*Ρ* < 0.01) after cerebral I/R were significantly increased, the expression of procaspase-1 and Cl.Caspase-1 at 3 (*Ρ* < 0.001) and 7 days (*Ρ* < 0.001) after reperfusion were increased and the levels of proIL-1β and mature IL-1β at 6 h, 24 h, 3 days, 7 days post-reperfusion were up-regulated (Fig. 2). Meanwhile, the expression of principal executioner of pyroptosis proteins GSDMD and GSDMD-N at 6 h, 24 h, 3 days, and 7 days after reperfusion were increased when compared with Sham group (Fig. 2). Above all, the expression levels of NLRP3 inflammasome such as NLRP3, procaspase-1, Cl.Caspase-1, GSDMD, GSDMD-N, proIL-1β and mature IL-1β showed a gradual increase at different time points after cerebral I/R injury and the contents showed the highest at 3 days after reperfusion. Hence, 3 days was selected as the interventional time point for further experiments.

### EA stimulus attenuated NLRP3 inflammasome mediated inflammatory reaction

**Fig. 3:**
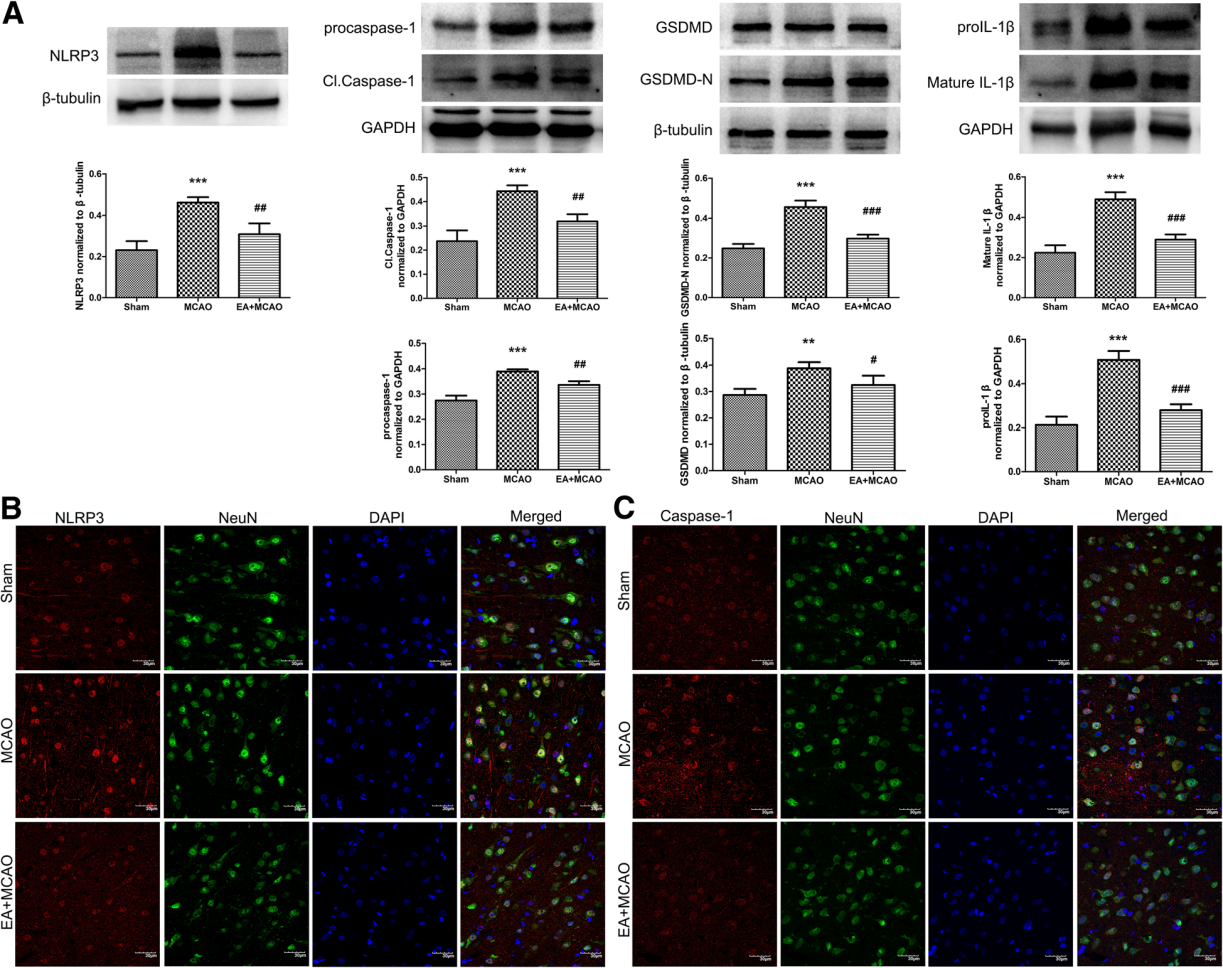
Effect of EA stimulus on the expression of NLRP3 inflammasome. **a** Representative western immunoblotting analysis of NLRP3, Caspase-1 and IL-1β (*n* = 4). EA stimulus significantly decreased the levels of NLRP3, procaspase-1, Cl.Caspase-1, GSDMD, GSDMD-N, proIL-1β and mature IL-1β when compared with MCAO group. **P < 0.01, ***P < 0.001 vs. Sham group, ^##^*P* < 0.01, ^###^*P* < 0.001 vs. MCAO group. (**b**, **c**) Immunofluorescent staining of NLRP3, Caspase-1, neuronal nuclei (NeuN), and nuclear marker DAPI. EA stimulus decreased the levels of NLRP3 positive neurons and Caspase-1 positive neurons when compared with MCAO group. Scale bar = 30 μm

To investigate the inhibitory effects of EA stimulus to the inflammatory response induced by NLRP3 inflammasome, the contents of NLRP3, Caspase-1, GSDMD and IL-1β were assessed by western blotting. Meanwhile, immunofluorescence (IF) staining was performed to observe the expressions of NLRP3 and Caspase-1. Compared with MCAO group, a significant decrease in the levels of NLRP3, procaspase-1, Cl.Caspase-1, GSDMD, GSDMD-N, proIL-1β and mature IL-1β was observed after EA stimulus (Fig. 3a). Immunohistochemical staining showed that NLRP3 and Caspase-1 were mainly co-localized with neuronal maker NeuN in the ischemic penumbra. Comparison of western blotting results demonstrated that NLRP3-positive neurons and Caspase-1-positive neurons in the EA + MCAO groups were less than those in MCAO group (Fig. 3b).

### Inhibition of NLRP3 inflammasome by EA stimulus was α7nAChR–dependent

**Fig. 4:**
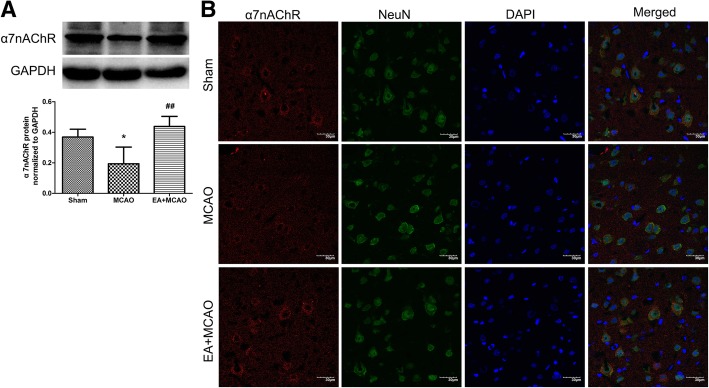
Effect of EA stimulus on the expression of α7nAChR. **a** Compared with sham group, the cerebral I/R injury decreased the contents of α7nAChR. Meanwhile, the EA up-regulated the contents of α7nAChR compared with MCAO group. *P < 0.05 vs. Sham group, ^##^*P* < 0.01 vs. MCAO group. **b** Representative immunofluorescent staining of α7nAChR, neuronal nuclei (NeuN), and nuclear marker DAPI. Cerebral I/R injury decreased the levels of α7nAChR-positive neurons compared with Sham group, while EA up-regulated the contents of α7nAChR-positive neurons when compared with MCAO group. Scale bar = 30 μm

To validate the participation of α7nAChR in the inhibition of NLRP3 inflammasome by EA, we used a specific α7nAChR antagonist α-BGT and a potent selective agonist for α7nAChR PHA-543,613. Western blotting was performed to detect the contents of α7nAChR and the levels of NLRP3, Caspase-1 and IL-1β (*n* = 4). Compared with sham group, cerebral I/R injury significantly decreased the contents of α7nAChR (*Ρ* < 0.05) (Fig. 4a). Meanwhile, the EA stimulus up-regulated the contents of α7nAChR when compared with MCAO group (*Ρ* < 0.01), (Fig. 4a). In addition, IF staining showed that α7nAChR was mainly co-localized with NeuN in the ischemic penumbra. In accordance with western blotting results, the α7nAChR-positive neurons in EA + MCAO groups were more than those in MCAO group (Fig. 4b).

**Fig. 5:**
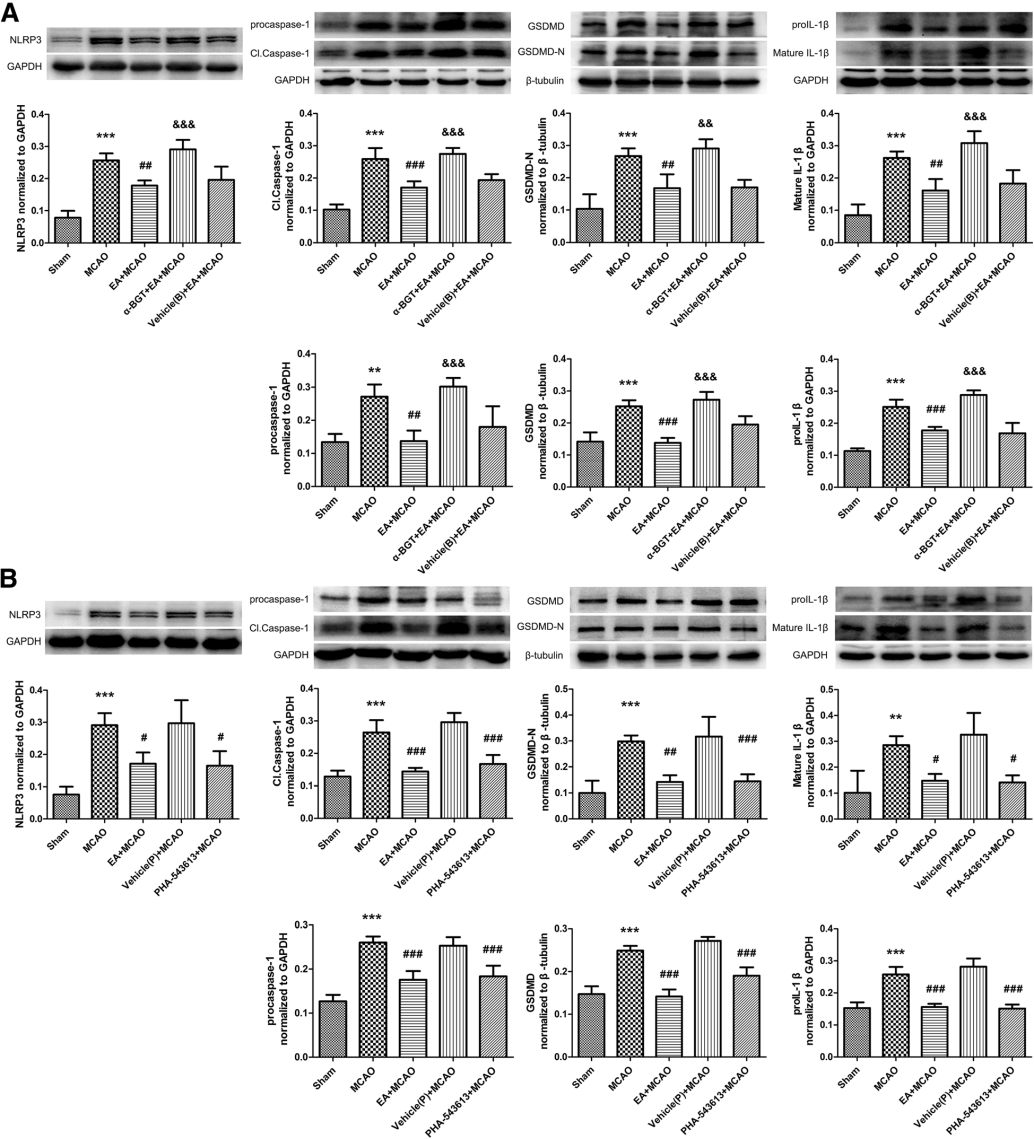
Inhibition of NLRP3 inflammasome by EA stimulus was α7nAChR–Dependent. **a** α7nAChR antagonist reversed the inhibition of NLRP3 inflammasome by EA stimulus. Representative western immunoblotting analysis of NLRP3, Caspase-1, GSDMD and IL-1β (*n* = 4). Compared with EA + MCAO group, the expression of NLRP3, procaspase-1, Cl.Caspase-1, GSDMD, GSDMD-N, proIL-1β and mature IL-1β were increased in α-BGT + EA + MCAO group. **P < 0.01, ***P < 0.001 vs. Sham group, ^##^*P* < 0.01, ^###^*P* < 0.001 vs. MCAO group, ^&&^*P* < 0.01, ^&&&^*P* < 0.001 vs. EA + MCAO group. **b** α7nAChR agonist mimicked the inhibition of NLRP3 inflammasome by EA stimulus. Representative western immunoblotting analysis of NLRP3, Caspase-1, GSDMD and IL-1β (*n* = 4). Comparison with MCAO groups showed that intraperitoneal injection of PHA-543,613 down-regulated the levels of NLRP3, procaspase-1, Cl.Caspase-1, GSDMD, GSDMD-N, proIL-1β and mature IL-1β. **P < 0.01, ***P < 0.001 vs. Sham group, ^#^*P* < 0.05, ^##^*P* < 0.01, ^###^*P* < 0.001 vs. MCAO group

When compared with EA + MCAO group, the expression of NLRP3, procaspase-1, Cl.Caspase-1, GSDMD, GSDMD-N, proIL-1β and mature IL-1β were significantly increased in α-BGT + EA + MCAO group (Fig. 5a). Comparison with MCAO groups showed that intraperitoneal injection of PHA-543,613 significantly down-regulated the levels of NLRP3, procaspase-1, Cl.Caspase-1, GSDMD, GSDMD-N, proIL-1β and mature IL-1β (Fig. 5b). So, EA could increase the expression of α7nAChR after cerebral I/R injury. The deficiency of α7nAChR induced by α-BGT reversed the inhibition of NLRP3 inflammasome by EA stimulus. PHA-543,613, an agonist of α7nAChR, mimicked the effect of EA stimulus and inhibited NLRP3 inflammasome mediated inflammatory response. These results suggested that the inhibition of NLRP3 inflammasome by EA stimulus was α7nAChR-dependent.

### EA stimulus regulated the balance between proinflammatory factors and anti-inflammatory cytokines through α7nAChR

**Fig. 6:**
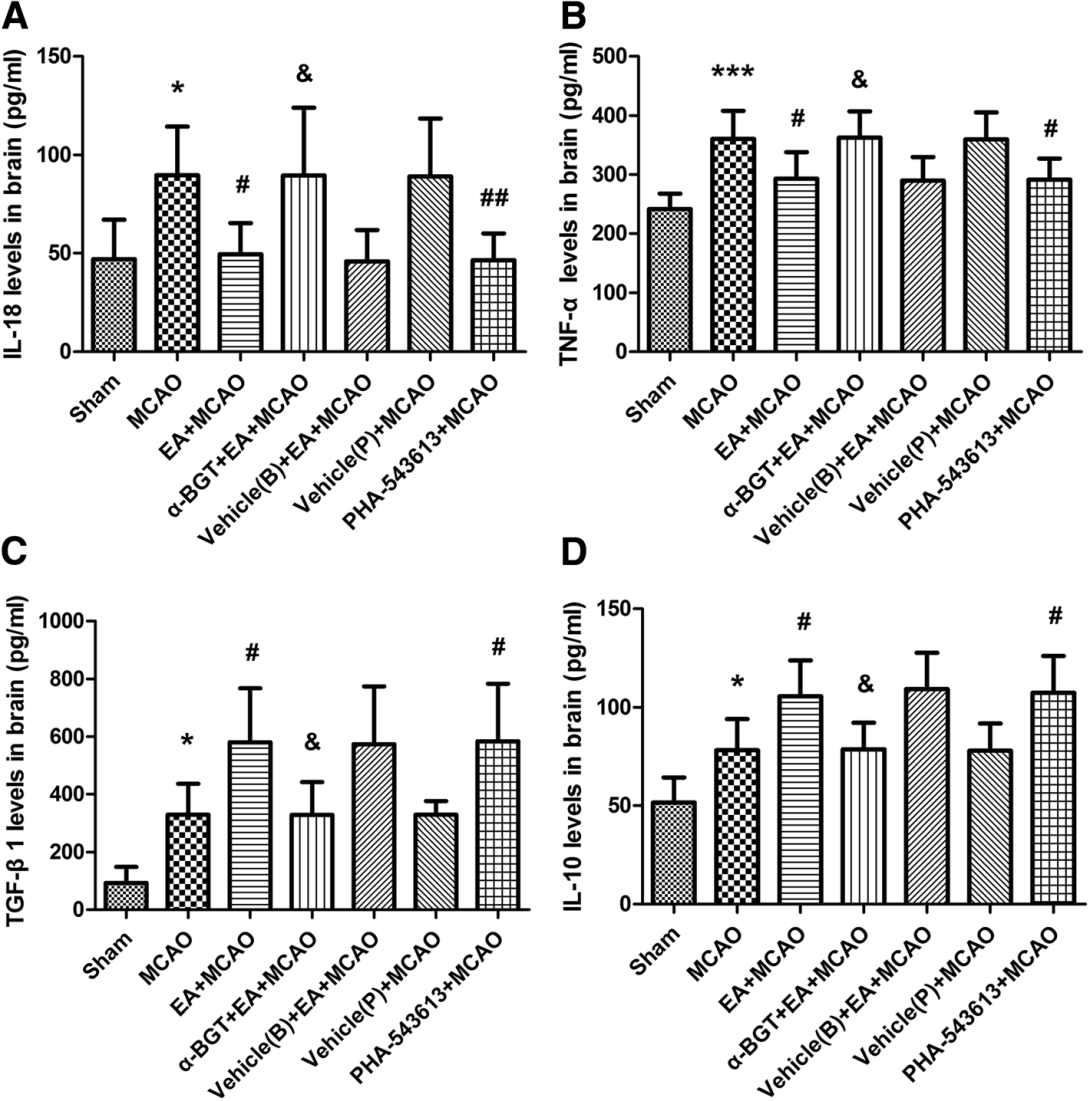
EA stimulus regulates the balance between proinflammatory factors and anti-inflammatory cytokines through α7nAChR. Representative ELISA analysis of proinflammatory factors IL-18 (**a**), TNF-α (**b**) and anti-inflammatory cytokines TGF-β1 (**c**), IL-10 (**d**) (*n* = 8 for each group). Compared with sham group, cerebral I/R injury up-regulated the levels of IL-18, TNF-α and TGF-β1, IL-10. EA stimulus down-regulated the levels of proinflammatory factors (IL-18, TNF-α) and up-regulated the contents of anti-inflammatory cytokines (TGF-β1, IL-10) as compared to MCAO group. Intraperitoneal injection of PHA-543,613 exerted similar beneficial effects as that of EA stimulus. Meanwhile, the administration of α7nAChR antagonist α-BGT reversed the effects of EA stimulus. *P < 0.05, ***P < 0.001 vs. Sham group, ^#^*P* < 0.05, ^##^*P* < 0.01 vs. MCAO group, ^&^*P* < 0.05 vs. EA + MCAO group

ELISA kits were used to evaluate the levels of IL-18, TNF-α and TGF-β1, IL-10 in the penumbral tissue to observe whether EA regulated the balance between proinflammatory factors and anti-inflammatory factors through α7nAChR. The results showed that the cerebral I/R injury up-regulated the levels of IL-18 (*Ρ* < 0.05), TNF-α (*Ρ* < 0.001) and the contents of TGF-β1 (*Ρ* < 0.05), IL-10 (*Ρ* < 0.05) when compared with sham group. Whereas, EA down-regulated the levels of IL-18 and TNF-α (*Ρ* < 0.05), and up-regulated the contents of TGF-β1 and IL-10 (*Ρ* < 0.05) when compared to MCAO group (Fig. 6). Comparison with EA + MCAO group, the expressions of IL-18 and TNF-α were significantly increased (*Ρ* < 0.05) and the contents of TGF-β1 and IL-10 were significantly decreased (*Ρ* < 0.05) in α-BGT + EA + MCAO group (Fig. 6). On the other hand, intraperitoneal injection of PHA-543,613 mimicked the effects of EA stimulus, down-regulated the levels of IL-18 (*Ρ* < 0.01) and TNF-α (*Ρ* < 0.05) and significantly up-regulated the contents of TGF-β1 and IL-10 (*Ρ* < 0.05) when compared with MCAO groups (Fig. 6).

### α7nAChR is involved in the neuroprotective effects of EA stimulus

**Fig. 7:**
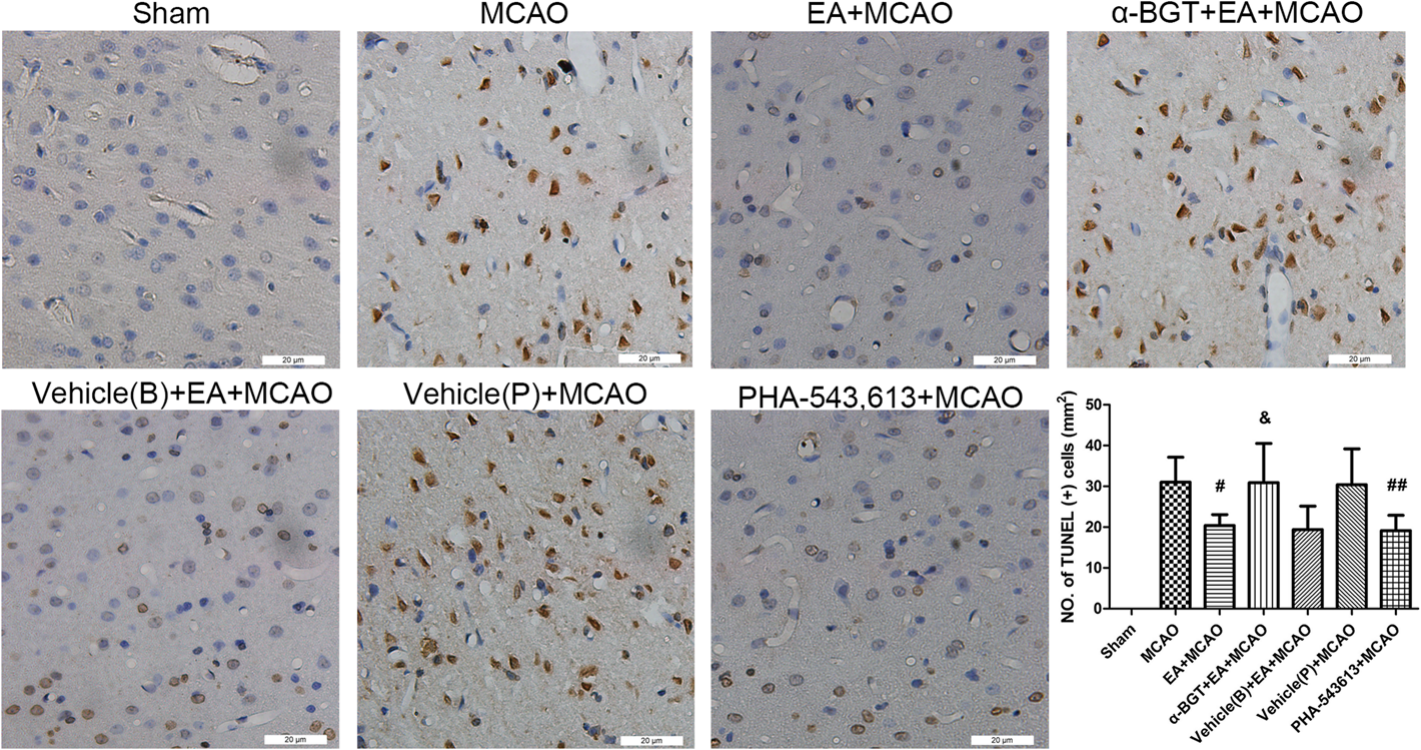
Cellular apoptosis at 3 days after reperfusion (*n* = 8). Representative TUNEL staining and the number of TUNEL positive cells. Compared with MCAO group, the cellular apoptosis was reduced in EA + MCAO group. When compared with EA + MCAO group, the cellular apoptosis was increased in α-BGT + EA + MCAO group. Intraperitoneal injection of PHA-543,613 reduced TUNEL positive cells when compared with MCAO groups. ^#^*P* < 0.05, ^##^*P* < 0.01 vs. MCAO group, ^&^*P* < 0.05 vs. EA + MCAO group; Bar = 20 μm

**Fig. 8:**
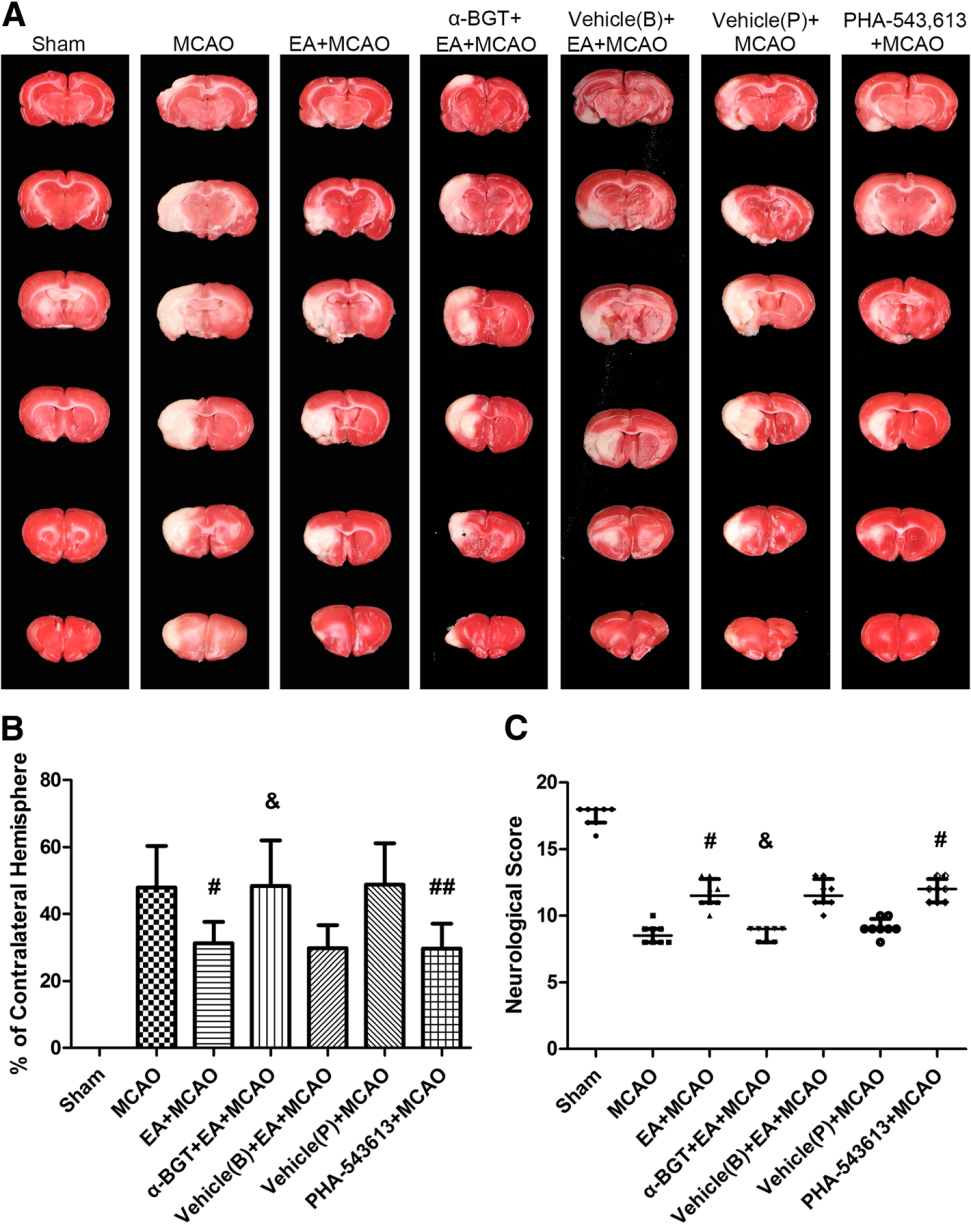
α7nAChR was involved in the neuroprotective effects of EA stimulus. Representative TTC staining of the cerebral infarct (**a**), infarct volume (**b**) and neurologic score (**c**) at 3 days after reperfusion (*n* = 8). *Red area* represents live tissue; and *white area* represents dead or dying tissue. The infarct volume of EA + MCAO group was significantly reduced and the neurological score was improved when compared with MCAO group. Compared with EA + MCAO group, the infarct volume of α-BGT + EA + MCAO group was significantly increased and neurological score was reduced. When compared with MCAO group, intraperitoneal injection of PHA-543,613 decreased the infarct size and alleviated neurological deficits. ^#^*P* < 0.05, ^##^*P* < 0.01 vs. MCAO group, ^&^*P* < 0.05 vs. EA + MCAO group

To check whether α7nAChR was involved in the neuroprotective effects of EA stimulus, the neurological deficit scores and infarct sizes were evaluated at 3 days after reperfusion (*n* = 8). TUNEL staining was conducted for measuring neuronal apoptosis (*n* = 8). Compared with MCAO group, the cellular apoptosis was reduced (*Ρ* < 0.05, Fig. 7), infarct volumes were decreased (*Ρ* < 0.05) and neurological scores were increased (*Ρ* < 0.05) in EA + MCAO group (Fig. 8). When compared with EA + MCAO group, the cellular apoptosis was increased (*Ρ* < 0.05, Fig. 7), infarct volumes were increased (*Ρ* < 0.05) and neurological scores were reduced (*Ρ* < 0.05) in α-BGT + EA + MCAO group (Fig. 8). Compared with MCAO groups, intraperitoneal injection of PHA-543,613 decreased the infarct sizes (*Ρ* < 0.01), produced neurological deficits (*Ρ* < 0.05, Fig. 8) and reduced TUNEL positive cells (*Ρ* < 0.01, Fig. 7). No differences were observed when compared with EA + vehicle (B) group and EA + MCAO group or the vehicle (P) group and the MCAO group. These results suggested that α7nAChR antagonist α-BGT could reverse the neuroprotective effects of EA stimulus and α7nAChR agonist PHA-543,613 mimics similar neuroprotective effects as that of EA stimulus.

## Discussion

Despite the decreased age-adjusted annual mortality rates of stroke worldwide for over the past two decades, the absolute number of incidences of first strokes, stroke survivors, stroke-related deaths, and global burden of stroke are more and increasing (Hankey 2017). “Prevention of Disease” is the basic theory of Chinese traditional medicine, and application of EA prior to the disease occurrence could modulate the body’s homeostasis, improve organ function and maintain the body in a balanced state (Lu et al. 2015). Meanwhile, perioperative acupuncture reduces the use of anesthetics and alleviates anesthesia-related complications, protects organs during the perioperative period, making it a promising approach to accelerate surgical recovery, especially in some specific populations, such as elderly patients and ‘triple-low’ patients. But the underlying mechanisms of these beneficial effects should be completely illustrated. It was first reported by our department that EA neuroprotection strategy has reduced the infarct volume and improved neurological outcome in the stroke model (Wang et al. 2009). Our previous researches have demonstrated that EA could increase the production of endocannabinoid AEA, 2-AG, CB1R (Wang et al. 2011) and adiponectin (APN), adiponectin receptor 1 (AdipoR1) (Guo et al. 2015), and regulate the RISK signaling pathway (ERKε, PKC, GSK-3β, STAT3), alleviating oxidative stress injury and inhibiting neuronal apoptosis (Guo et al. 2015; Sun et al. 2016; Wei et al. 2014; Zhou et al. 2013). According to the clinical practice guidelines on the management of postoperative pain from the American Pain Society, clinicians consider transcutaneous electrical nerve stimulation (TENS) as an adjunct to other postoperative pain treatments (weak recommendation, moderate-quality evidence) (Chou et al. 2016). Though accumulated evidences from clinical trials and updated reviews support its effects in perioperative and stroke rehabilitation (Vickers et al. 2018), its mechanism in regulating the inflammation after cerebral I/R injury remains unclear (Ernst et al. 2011; Goldman et al. 2010).

Initiation of inflammatory reaction involves a cytosolic multiprotein complexes that are termed as ‘inflammasomes’ (Walsh et al. 2014), which function as intracellular sensors for PAMP and DAMP, and act as infectious agents or disease-associated host molecules (Walsh et al. 2014). Assembly of inflammasome activates pro-inflammatory caspases, mainly caspase-1, leading to the cleavage and release of IL-1β and IL-18, as well as pyroptotic cell death (Savage et al. 2012). Pyroptosis is characterized by rapid plasma membrane rupture, cellular swelling, release of pro-pyroptosis factors, DNA cleavage and nuclear condensation, based on cleaved caspase-1 through an unknown mechanism (Bergsbaken et al. 2009). The pore-forming protein gasdermin D (GSDMD) that is cleaved by caspase-1 was identified as the principal executioner of pyroptosis. Caspase-1 and GSDMD can promote the activation of IL-1β and IL-18 by directly or through some signaling cascade, leading to pyroptosis (McKenzie et al. 2018).

NLRP3 inflammasome acts as a general sensor of cellular damage or stress, and is noted for its broad array of activating stimuli, such as endogenous danger signals (extracellular ATP, mROS, mtDNA, ER stress etc), bacterial, fungal and viral components (Walsh et al. 2014). In the current study, we observed that cerebral I/R injury increased the expression of NLRP3 inflammasome proteins and the principal executioner of pyroptosis proteins in rats at 6 h remained high for at least 7 days following reperfusion, among which the highest contents were observed at 3 days after reperfusion. Recent studies have implicated that NLRP3 inflammasomes were activated in the microglial cells soon after the onset of cerebral I/R injury and then were expressed in neurons and microvascular endothelial cells later, but were mainly appeared in neurons (Gong et al. 2018). Thus, we focused on the levels of NLRP3 inflammasome in neurons on day 3 following reperfusion. Interestingly, EA stimulus showed convincing protective effects by reducing the levels of NLRP3 inflammasome in neurons. This was consistent with the previous studies, which demonstrated the detection of NLRP3 inflammasome in the cytoplasm of cerebral cortical neurons and was associated with the incidence and progression of ischemic stroke (Compan et al. 2012; Yang et al. 2014).

Cytokines production by immune system contributes to the pathophysiologic mechanisms of cerebral I/R injury. IL-1β and TNF-α are the key initiators of inflammation, making important contributions to cellular activation and cytokines production. IL-18 is considered as a pivotal regulator of interferon-γ responses in NK cells and T cells. IL-10 and TGF-β produced by reactive glial cells and infiltrating immune cells acts as major anti-inflammatory molecules in ischemic brain. It is proved that viral overexpression of IL-10 had neuroprotective effects after cerebral ischemia (Ooboshi et al. 2005) and TGF-β had anti-inflammatory effects by inhibiting excessive neuroinflammation during the subacute phase of stroke (Cekanaviciute et al. 2014). In CNS, the microglia, astrocytes and neurons express receptors for these cytokines, participating in systemic inflammatory responses (Walsh et al. 2014).

In the nervous system, cholinergic anti-inflammatory pathway, which is an efferent neural signaling pathway of vagus nerve inflammatory reflex, plays an essential role in the inhibition of cytokines release, thereby preventing tissue injury and death (Bertrand et al. 2015). Among these nicotinic receptors, α7nAChR is considered to be the most important receptor for transmitting cholinergic anti-inflammatory signals (Han et al. 2017; Wang et al. 2003). It is reported that vagus nerve stimulation or administration of α7nAChR agonists suppressed proinflammatory cytokines (such as TNF-α, IL-1β, IL-6, IL-8 and HMGB1) (Hoover 2017; Wang et al. 2004). Mice with genetic knockout of α7nAChR subunit or vagotomy when exposed to endotoxins exhibited an unbalanced, excessive inflammatory response characterized by exaggerated TNF levels (Borovikova et al. 2000; Wang et al. 2003). Moreover, the stimulation of vagus nerve in α7nAChR-ko animals failed to suppress cytokines synthesis, but significantly inhibited cytokines release in wild-type littermates (Wang et al. 2003). Signal transduction of α7nAChR in neurons is modulated by ligand-gated ion channel and ligand-receptor interaction on immune cells, reduced nuclear translocation of NF-κB as well as activation of the transcription factors STAT3 via JAK2 phosphorylation (de Jonge et al. 2005; Wang et al. 2004). This study indicated that α7nAChR-associated cholinergic signals function as a governor on neurological modulation of cytokines synthesis and limited the magnitude of immune response. Thus, we hypothesized that α7nAChR may be involved in the neuroprotective effects of EA by inhibiting NLRP3 inflammasome.

Recent studies have implicated the involvement of α7nAChR in neuronal survival and synaptic plasticity, including anti-inflammation and anti-apoptosis. It was significantly decreased during stroke and EA attenuated the impairment of central cholinergic system induced by ischemia (Chi et al. 2018). Consistently, we found that the expression of α7nAChR was decreased after reperfusion, whereas EA up-regulated the contents of α7nAChR in neurons after cerebral ischemia. To further test this hypothesis, a selective α7nAChR agonist PHA-543,613 was administrated prior to MCAO. Meanwhile, a specific α7nAChR antagonist α-BGT was injected 0.5 h prior to EA stimulus via intracerebroventricularly. The results showed that EA stimulus that down-regulated the levels of IL-18 and TNF-α, and up-regulated the contents of TGF-β1 and IL-10, regulated the balance between proinflammatory factors and anti-inflammatory cytokines via α7nAChR. More interestingly, it showed that PHA-543,613 mimicked the anti-inflammatory potential of EA and exerted similar protective effects as of EA, which not only inhibited the expression of NLRP3, procaspase-1, Cl.Caspase-1, GSDMD, GSDMD-N, proIL-1β and mature IL-1β, but also decreased the infarct size, alleviating neurological deficits and reducing cellular apoptosis. Also, the anti-inflammatory and neuroprotective effects of EA were reversed by using α-BGT. Above all, our results provided persuasive evidence supporting that EA stimulus exhibited neuroprotective effects by a dependent mechanism, at least or in part, on the inhibition of NLRP3 inflammasome via α7nAChR.

## Conclusions

**Fig. 9:**
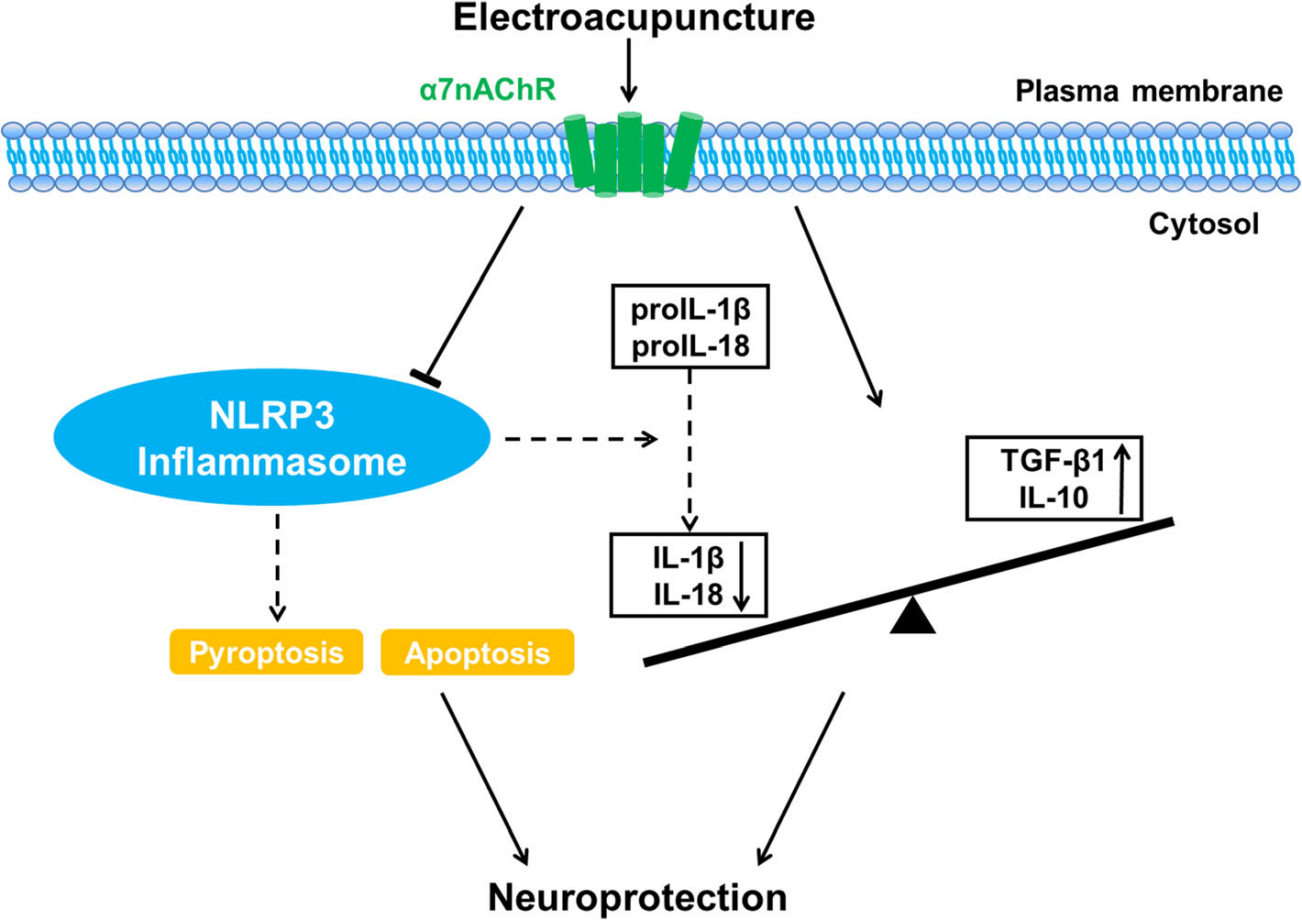
Schematic diagram illustrating the mechanisms of EA-induced neuroprotection. Electroacupuncture attenuated cerebral ischemic injury and neuroinflammation through α7nAChR-mediated inhibition of NLRP3 inflammasome in stroke rats

Our research highlighted that EA stimulus produced considerable neuroprotective effects by up-regulating α7nAChR in neurons, subsequently inhibiting NLRP3 inflammasome associated inflammatory response, reducing cellular apoptosis and regulating the balance between proinflammatory factors and anti-inflammatory cytokines after transient cerebral ischemic injury (Fig. 9). Although further studies are warranted for improving the understanding of the signal transduction pathways involved between the cholinergic anti-inflammatory system and inflammasome, our current findings established a novel mechanism of α7nAChR-dependent regulation of NLRP3 inflammasome that was induced by EA stimulus. This research supported the potent clinical application of activating α7nAChR-dependent cholinergic anti-inflammatory system and inhibiting inflammasome to defend ischemic injury, providing sufficient basic proof for the clinical translation of EA.

## Abbreviations

Caspase-1: Cysteine aspartate specific Proteinases-1
CB1R: Cannabinoid receptor 1
DAMPs: Danger-associated molecular patterns
EA: Electroacupuncture
ELISA: Enzyme-linked immunosorbent assay
HMGB1: High mobility group box 1
I/R: Ischemia/reperfusion
IL-1β: Interleukin-1β
IRFs: Interferon regulatory factors
MCAO: Middle cerebral artery occlusion
NeuN: Neuron nucleus
NLRs: Nucleotide-binding domain and leucine-rich repeat-containing
PAMPs: Pathogen-associated molecular patterns
PRRs: Pattern-recognition receptors
RISK: Reperfusion Injury Salvage Kinase
ROS: Reactive oxygen species
TGF-β1: Transforming growth factor-β1
TLRs: Toll-like receptors
TNF-α: Tumor necrosis factor-α
t-PA: Tissue-type plasminogen activators
TUNEL: Terminal deoxynucleotidyl transferase-mediated dUDP-biotin nick end labeling
α7nAChR: α 7 nicotinic acetylcholine receptor
α-BGT: α-bungarotoxin
